# Risk factors for intertrochanteric femoral fractures with concomitant lateral wall involvement in elderly women

**DOI:** 10.3389/fsurg.2025.1730094

**Published:** 2025-12-18

**Authors:** Gaolong Shi, Zhenghui Hu, Zonggang Xie, Jun Gu, Qiyan Lu, Zhuoyan Ling

**Affiliations:** Department of Orthopedics, The Second Affiliated Hospital of Soochow University, Suzhou, China

**Keywords:** intertrochanteric fractures, femoral lateral wall fractures, bone mineral density, body mass index, bone turnover markers

## Abstract

**Background:**

Intertrochanteric femoral fractures are common osteoporotic injuries in elderly women, and disruption of the lateral femoral wall has been recognized as a key factor contributing to fracture instability and fixation failure. Low bone mineral density (BMD), impaired bone metabolism, and decreased body mass index (BMI) are known to influence fracture patterns in the elderly; however, the specific factors predisposing patients to concomitant lateral wall involvement remain unclear. Therefore, the purpose of this study was to compare BMD, bone turnover markers, BMI, and age between elderly women with isolated intertrochanteric fractures and those with additional lateral wall involvement, and to identify independent risk factors associated with lateral wall fractures.

**Methods:**

Between January 2023 and February 2024, 49 postmenopausal women with intertrochanteric femoral fractures were treated at our institution. Among them, 29 had isolated intertrochanteric fractures and 20 sustained concomitant lateral wall fractures. Independent-samples *t* tests were used to compare age, BMI, and BMD between groups, whereas Mann–Whitney *U* tests were applied for P1NP and β-CTX. Binary logistic regression was performed with age, BMI, lumbar BMD, and hip BMD as covariates to assess their associations with lateral wall involvement.

**Results:**

Significant differences were observed between groups in age, BMI, lumbar BMD, and hip BMD, whereas P1NP and β-CTX showed no significant differences. Logistic regression revealed that lower BMI, lumbar BMD, and hip BMD were independently associated with the occurrence of lateral wall fractures.

**Conclusion:**

Low BMI and decreased lumbar and hip BMD are significant risk factors for lateral wall involvement in intertrochanteric femoral fractures among elderly women.

**Trial Registration:**

ClinicalTrials.gov identifier: NCT07196982.

## 1 Introduction

Intertrochanteric fractures are among the most common hip fractures, and their incidence continues to rise with population aging. The mean age at presentation is approximately 70 years, accounting for nearly 21% of all fractures in the elderly, with a female predominance (male-to-female ratio ≈ 1:3). Given that intertrochanteric fractures predominantly occur in elderly patients, who frequently have osteoporosis and comorbidities such as hypertension and diabetes, nonoperative management with prolonged bed rest carries a high risk of complications and mortality. Consequently, surgical fixation has become the standard of care. Current debates surrounding the surgical management of intertrochanteric fractures include the choice between intramedullary and extramedullary fixation, optimal timing of surgery, internal fixation versus arthroplasty, ideal placement of head–neck screws (including tip–apex distance), management of the lesser trochanter, and strategies for concomitant lateral wall fractures. Intramedullary nailing has gradually become the gold standard for intertrochanteric fracture fixation (1). However, because intramedullary devices do not reestablish medial cortical support of the proximal femur, the risk of fixation failure persists in unstable fracture patterns (2). In particular, AO/OTA 31-A3.3 reverse oblique intertrochanteric fractures with compromised lateral wall integrity subject the fixation construct to excessive stress, leading to a markedly increased risk of secondary displacement or implant failure (3).

Since Gotfried introduced the concept of the lateral wall in 2004, increasing attention has been directed toward intertrochanteric fractures with lateral wall involvement. It is now widely recognized that, beyond the posteromedial fragment involving the lesser trochanter and calcar femorale, the integrity of the lateral wall is a critical determinant of intertrochanteric fracture stability. Lateral wall fracture has been identified as an independent risk factor for fixation failure in intertrochanteric fractures (4, 5). Lateral wall involvement may impair gluteus medius function, thereby reducing lower limb balance and stability (6). An intact lateral wall provides critical buttress support to the proximal fragment in unstable intertrochanteric fractures, whereas disruption of the lateral wall predisposes to excessive collapse and varus malalignment of the hip (4, 7). Patients with lateral wall fractures often require delayed postoperative weight bearing, which markedly limits independence and mobility. Prolonged bed rest, although intended to prevent nonunion, increases the likelihood of medical complications after hip fracture in elderly patients. Inadequate intraoperative management of lateral wall fractures can compromise postoperative limb function and, in severe cases, result in implant failure or loss of fixation.

The definition of the lateral wall has evolved over time. Gotfried initially described it simply as a proximal extension of the femoral shaft (4). In 2007, Palm (5) further refined the concept, defining its proximal boundary as the vastus lateralis ridge. In 2014, Haq (8) proposed that the superior boundary of the lateral wall corresponds to the intersection of a tangent drawn along the superior border of the femoral neck with the greater trochanter, whereas the inferior boundary is defined by the intersection of a tangent along the inferior border of the femoral neck with the lateral cortex of the femur. The segment of cortex between these two reference lines constitutes the lateral wall. Zhang et al. (9) suggested that the superior boundary of the lateral wall should be the vastus lateralis ridge, whereas the inferior boundary should be defined by the intersection of a horizontal plane through the inferior margin of the lesser trochanter with the lateral femoral cortex. At present, most domestic scholars favor Zhang's definition of the lateral wall, particularly in relation to fracture classification systems.

Elderly women are at substantially higher risk of osteoporotic fractures than men because of accelerated bone loss after menopause. Consequently, intertrochanteric fractures in elderly women represent the most common type of hip fracture and warrant particular clinical attention. Lateral wall involvement carries unique clinical implications in intertrochanteric fractures, and inadequate management often leads to suboptimal outcomes. The presence or absence of lateral wall disruption is therefore closely associated with treatment efficacy. Preoperative evaluation of lateral wall integrity is essential for selecting the most appropriate fixation strategy and implant.

Therefore, we conducted a retrospective study of patients with intertrochanteric fractures and concomitant lateral wall involvement treated at our institution between 2023 and 2024, aiming to identify potential etiological factors and risk factors associated with these fractures. Findings from this study may help improve preoperative planning in high-risk patients, including the use of CT to avoid missed diagnoses, and provide guidance for optimizing treatment and prevention strategies in this population. We hypothesized that lower bone mineral density and reduced body mass index would be independently associated with an increased likelihood of concomitant lateral wall involvement in intertrochanteric femoral fractures.

## 2 Methods

This was a retrospective case–control study (Level of Evidence III) involving postmenopausal women with intertrochanteric femoral fractures treated at the Second Affiliated Hospital of Soochow University between January 2023 and February 2024.

**Figure 1:**
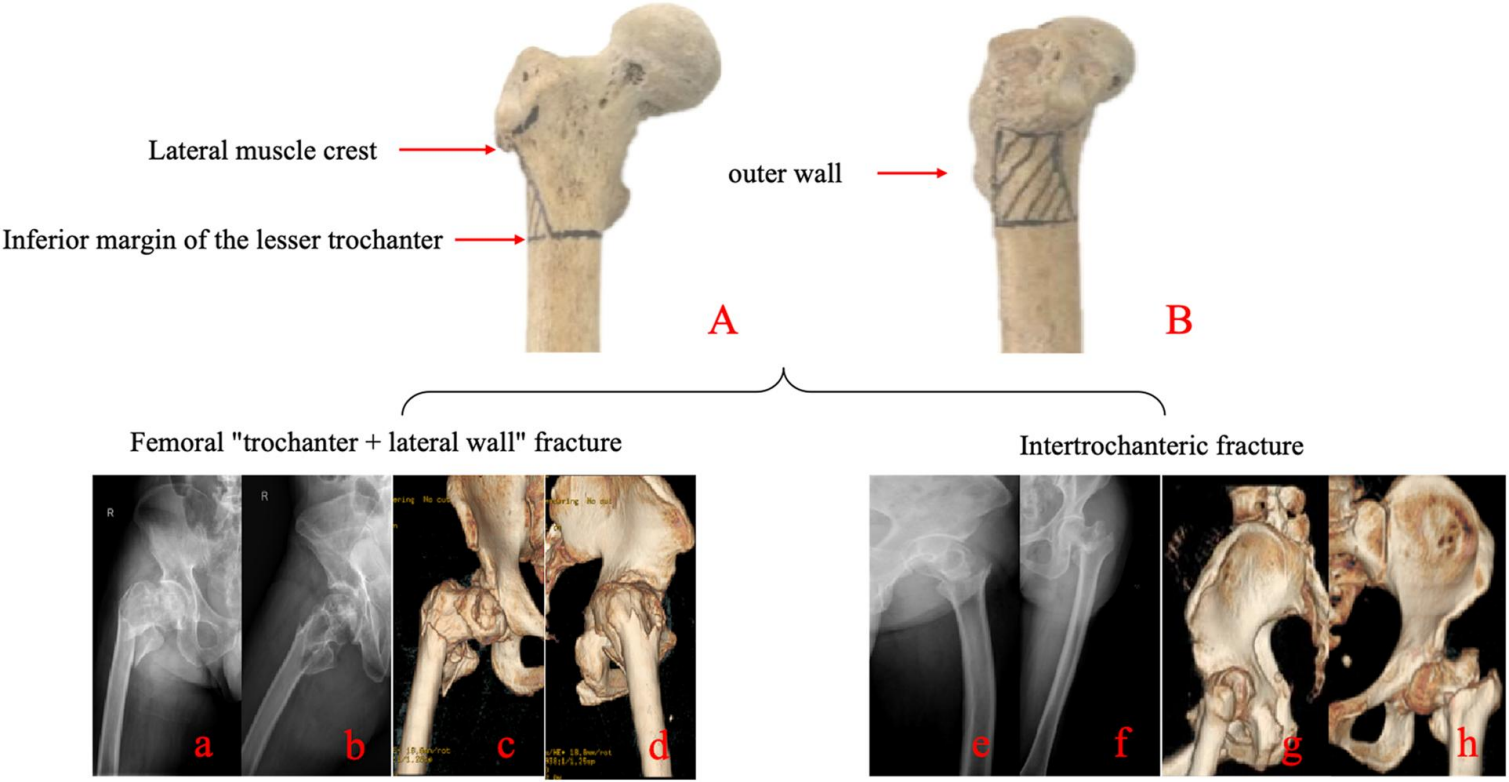
Definition of the lateral femoral wall and comparative imaging of intertrochanteric fractures with and without lateral wall involvement. **(A)** The superior boundary of the lateral wall is defined by the vastus lateralis ridge (lateral muscle crest), and the inferior boundary by the inferior margin of the lesser trochanter. **(B)** Highlighted segment of the lateral femoral wall. Bottom panels: Radiographic examples. Left side: Intertrochanteric fracture with lateral wall involvement, shown in x-ray images (a,b) and CT three-dimensional reconstructions (c,d). Right side: Isolated intertrochanteric fracture without lateral wall involvement, shown in x-ray images (e,f) and CT three-dimensional reconstructions (g,h).

**Figure 2:**
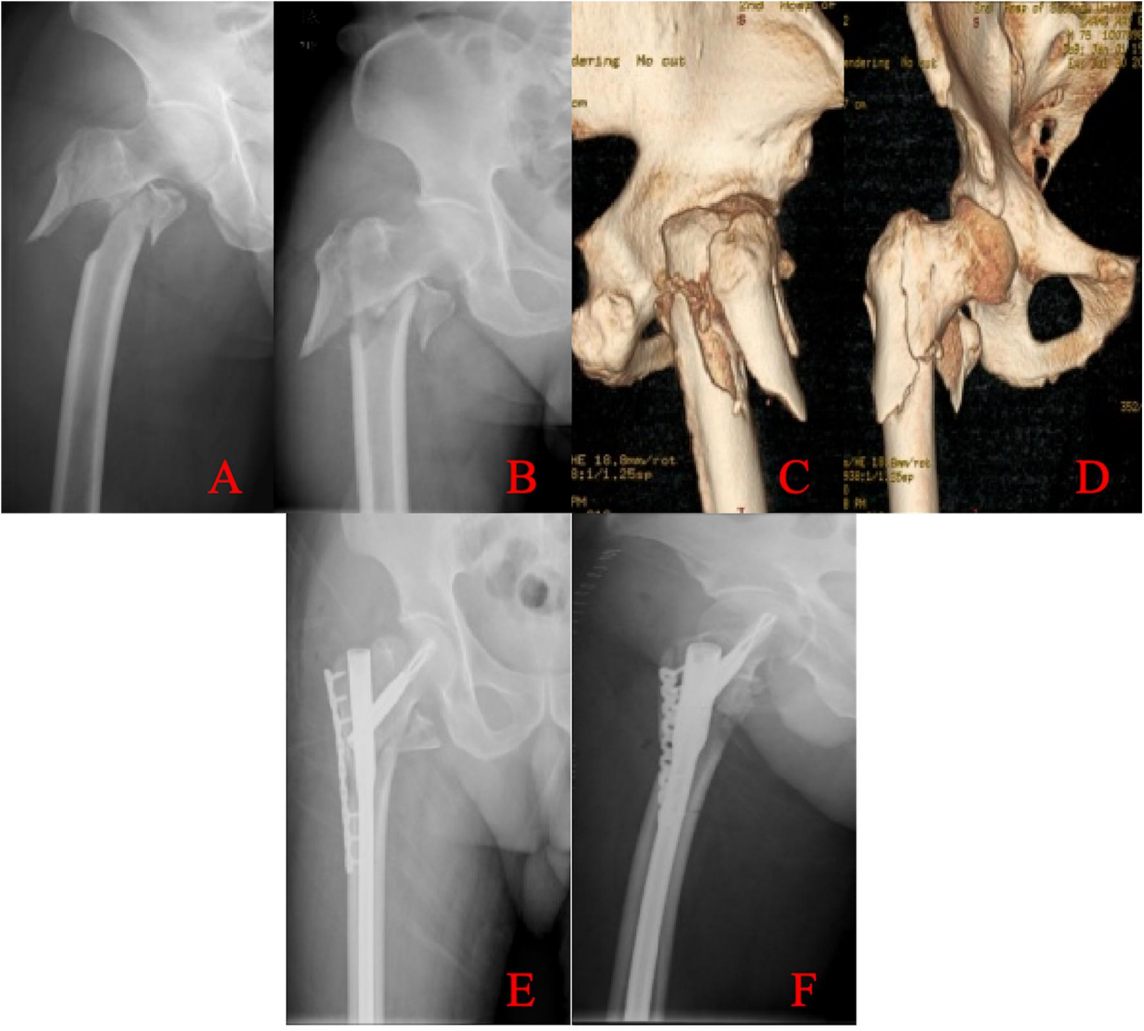
Radiographic images of a typical case of proximal intramedullary nail-assisted lateral plate fixation for an intertrochanteric fracture with lateral wall involvement. Top panels: Preoperative images. **(A,B)** x-ray images demonstrating the intertrochanteric fracture with lateral wall involvement. **(C,D)** Three-dimensional CT reconstructions illustrating the fracture morphology. Bottom panels: Postoperative images. **(E,F)** x-ray images showing fixation with a proximal femoral nail antirotation (PFNA) combined with an adjunctive lateral trochanteric plate.

A total of 152 postmenopausal women with intertrochanteric femoral fractures were initially screened from the Osteoporotic Fracture Unit of the Department of Joint Surgery at our institution between January 2023 and February 2024. According to the aforementioned inclusion and exclusion criteria, 49 patients were eligible and included in the final analysis. Fracture classification was performed by senior orthopedic surgeons based on preoperative CT and radiographic findings. Fracture classification was performed independently by two senior orthopedic trauma surgeons using preoperative radiographs and CT images. In cases of disagreement, a consensus was reached through joint review and discussion. The interobserver agreement for fracture classification and identification of lateral wall involvement was assessed using Cohen's kappa coefficient; the kappa value demonstrated substantial agreement (*κ* = 0.72). Lateral wall fractures were diagnosed using the following criteria: the superior boundary was defined as the vastus lateralis ridge, and the inferior boundary as the intersection of a horizontal plane through the inferior margin of the lesser trochanter with the lateral femoral cortex (Figure 1). The 49 patients were stratified into two groups: an isolated intertrochanteric fracture group (*n* = 29) and an intertrochanteric fracture with lateral wall involvement group (*n* = 20). This study has been approved by the Ethics Committee of the Second Affiliated Hospital of Soochow University with the ethics number: JD-HG-2025-107. This clinical trial was registered at ClinicalTrials.gov (Identifier: NCT07196982). This study was a retrospective case analysis. Surgical methods were extracted from operative records and confirmed by postoperative imaging. According to group allocation, patients with intertrochanteric femoral fractures underwent closed reduction and internal fixation using a proximal femoral nail antirotation (PFNA). Patients with “intertrochanteric + lateral wall” femoral fractures underwent open reduction and internal fixation with PFNA combined with lateral femoral wall plate fixation (Figure 2).

Inclusion criteria: (1) postmenopausal women diagnosed with intertrochanteric femoral fractures; (2) history of low-energy trauma; (3) surgical fixation with a proximal femoral nail antirotation (PFNA) device, with or without an adjunctive lateral trochanteric plate; (4) complete demographic and clinical data, including age, height, weight, bone mineral density (BMD), and bone turnover markers; (5) eligibility for retrospective case–control analysis.

Exclusion criteria: (1) high-energy trauma (e.g., motor vehicle accidents); (2) severe systemic comorbidities, including hepatic or renal failure, bone tumors, or endocrine disorders such as hyperparathyroidism; (3) use of medications known to affect bone metabolism within 6 months preoperatively; (4) diagnosis of diabetes mellitus; (5) history of chronic smoking or alcohol abuse; (6) inability to provide accurate anthropometric data (height and weight).

The study parameters included patient age, height, weight, body mass index (BMI), bone turnover markers, and BMD.

1. Patient age, height, and weight were documented, and BMI was calculated as weight in kilograms divided by height in meters squared. Because direct measurement was not feasible at admission due to hip fracture, height and weight data were extracted from medical records and subsequently verified by telephone follow-up to ensure accuracy.
2. Bone turnover markers, including N-terminal propeptide of type I procollagen (P1NP) and β-isomerized C-terminal telopeptide of type I collagen (β-CTX), were measured after admission. Serum samples for P1NP and β-CTX were collected under standardized conditions. All patients underwent fasting blood draws in the early morning (between 6:30 and 8:00 AM) on the first postoperative day to minimize circadian variation and postprandial fluctuations. Samples were immediately processed and analyzed using standardized laboratory protocols.
3. After internal fixation, patients underwent BMD assessment in the densitometry unit using dual-energy x-ray absorptiometry (DXA; GE Healthcare, USA) of the contralateral hip and lumbar spine. Both T-scores and site-specific BMD values were recorded. Lumbar spine measurements included L1, L2, L3, L4, L1–4, and L2–4, whereas hip measurements included the femoral neck, Ward's triangle, and the greater trochanter.

### 2.1 Statistical analysis

Data were analyzed using SPSS software (version 21.0; IBM Corp., Armonk, NY, USA). Continuous variables were expressed as mean ± standard deviation (SD) and compared between groups using independent-samples *t* tests. A two-tailed *P* value < 0.05 was considered statistically significant. Serum P1NP and β-CTX, which were not normally distributed, were analyzed using Mann–Whitney *U* tests for two independent samples and reported as medians with interquartile ranges (IQRs). Statistical significance was set at *P* < 0.05. Prior to logistic regression, multicollinearity among age, BMI, lumbar spine BMD, and hip BMD was evaluated using variance inflation factors. All VIFs fell within acceptable limits (VIF < 5), indicating no evidence of concerning collinearity that would invalidate model estimates. Age, BMI, lumbar spine BMD, and femoral neck BMD were included as independent variables in binary logistic regression models to evaluate their associations with isolated intertrochanteric fractures versus those with lateral wall involvement. A *P* value < 0.05 was considered statistically significant.

## 3 Results

### 3.1 Patient age and lateral wall involvement

Of the 49 patients included, the mean age was 72.9 ± 10.7 years in the isolated intertrochanteric fracture group (*n* = 29) and 78.9 ± 9.2 years in the group with concomitant lateral wall involvement (*n* = 20). Independent-samples t tests demonstrated that patients with lateral wall involvement were significantly older than those with isolated intertrochanteric fractures (*P* < 0.05; Table 1).

**Table 1:** Comparison of age of patients with intertrochanteric fractures and intertrochanteric + lateral wall fractures.

Fracture type	Number of cases	Age (years, Means ± SD)
Intertrochanteric fracture	29	72.86 ± 10.69
Intertrochanteric + lateral wall fracture	20	78.85 ± 9.16
*P* value	-	0.047

### 3.2 BMI and lateral wall involvement

The mean BMI was 24.0 ± 3.2 in the isolated intertrochanteric fracture group (*n* = 29) and 21.6 ± 2.7 in the group with concomitant lateral wall involvement (*n* = 20). Independent-samples *t* tests revealed that patients with lateral wall involvement had significantly lower BMI values compared with those with isolated intertrochanteric fractures (*P* < 0.05; Table 2).

**Table 2:** Comparison of BMI index between patients with intertrochanteric fracture and intertrochanteric + lateral wall fracture.

Fracture type	Number of cases	BMI index (kg/m^2^, Means ± SD)
Intertrochanteric fracture	29	23.95 ± 3.21
Intertrochanteric + lateral wall fracture	20	21.57 ± 2.69
*P* value	-	0.009

BMI index, body mass index.

### 3.3 Bone turnover markers and lateral wall involvement

Serum P1NP and β-CTX values were not normally distributed in either group; therefore, data were analyzed using the Mann–Whitney *U* test and reported as medians with interquartile ranges (IQRs). In the isolated intertrochanteric fracture group (*n* = 29), median (IQR) serum P1NP and β-CTX levels were 48.2 (34.0–89.3) and 462.4 (322.7–676.0), respectively. In the group with concomitant lateral wall involvement (*n* = 20), median (IQR) serum P1NP and β-CTX levels were 49.1 (35.3–74.8) and 591.9 (423.3–695.6), respectively. Both comparisons yielded *P* values > 0.05 (Table 3), indicating no statistically significant differences between groups.

**Table 3:** Comparison of serum parameters in patients with intertrochanteric fracture and intertrochanteric + lateral wall fracture.

Fracture type	Number of cases	P1NP	β-CTX
		P50 (P25, P75)	P50 (P25, P75)
Intertrochanteric fracture	29	48.17 (34.04, 89.27)	462.40 (322.70, 676.00)
Intertrochanteric + lateral wall fracture	20	49.08 (35.29, 74.75)	591.85 (423.33, 695.60)
*P* value	-	0.95	0.645

P1NP, amino-terminal peptide of procollagen type 1; β-CTX, degradation product of β-collagen.

### 3.4 Bone mineral density and lateral wall involvement

All patients underwent postoperative BMD assessment. Lumbar spine and overall hip BMD values were significantly lower in patients with concomitant lateral wall involvement than in those with isolated intertrochanteric fractures (*P* < 0.05; Table 4). By contrast, no significant differences were detected in BMD values at the femoral neck, Ward's triangle, or greater trochanter (*P* > 0.05; Table 4).

**Table 4:** Comparison of BMD at different sites in patients with intertrochanteric fracture and intertrochanteric + lateral wall fracture.

Observational indicators	Model 1		Model 2	
	Risk OR	*P* value	OR	*P* value
Age	2.348 (0.423, 13.047)*	0.329	–	–
BMI	0.268 (0.072, 0.998)*	.049	–	–
Lumbar spine BMD	5.114 (1.293, 20.221)*	.02	4.856 (1.081, 21.808)^#^	.039
Hip BMD	8.667 (1.969, 38.151)*	.048	5.604 (1.082, 29.025)^#^	.045

*P < 0.05 in Model 1. ^#^*P* < 0.05 in Model 2.

### 3.5 Logistic regression analysis of risk factors

Logistic regression was performed on the four variables that demonstrated significant group differences (age, BMI, lumbar spine BMD, and hip BMD). Results showed that lower BMI was significantly associated with an increased risk of lateral wall involvement in intertrochanteric fractures (*P* < 0.05; Table 5). Similarly, reduced lumbar spine and hip BMD values were significant risk factors (*P* < 0.05; Table 5). In contrast, age was not independently associated with fracture type (*P* > 0.05; Table 5). Because age and BMI are known determinants of BMD, these variables were excluded from further analysis. Univariate regression demonstrated that decreased BMD was an independent risk factor for lateral wall involvement.

**Table 5:** Logistic regression analysis results of age, BMI, and bone density in intertrochanteric fractures and intertrochanteric + lateral wall fractures.

Fracture type	Intertrochanteric fracture (29 cases)	Intertrochanteric + lateral wall fracture (20 cases)	*P* value
Lumbar Spine			
Lumbar spine BMD (T-score, Mean ± SD)	−1.41 ± 0.99	−2.22 ± 0.94	.007
Lumbar vertebra 1 (g/cm^2^, Mean ± SD)	0.84 ± 0.13	0.80 ± 0.15	0.334
Lumbar vertebra 2 (g/cm^2^, Mean ± SD)	0.94 ± 0.18	0.84 ± 0.12	0.058
Lumbar vertebra 3 (g/cm^2^, Mean ± SD)	0.99 ± 0.17	0.90 ± 0.14	0.054
Lumbar vertebra 4 (g/cm^2^, Mean ± SD)	1.02 ± 0.17	0.90 ± 0.18	.032
Lumbar vertebra 1–4 (g/cm^2^, Mean ± SD)	0.95 ± 0.12	0.87 ± 0.14	0.051
Lumbar vertebra 2–4 (g/cm^2^, Mean ± SD)	0.98 ± 0.13	0.89 ± 0.14	.032
Hip			
Hip BMD (T-score, Mean ± SD)	−1.23 ± 1.08	−2.11 ± 1.35	.015
Femoral Neck (g/cm^2^, Mean ± SD)	0.77 ± 0.11	0.69 ± 0.17	0.079
Wards' Triangle (g/cm^2^, Mean ± SD)	0.59 ± 0.17	0.58 ± 0.22	0.794
Greater Trochanter (g/cm^2^, Mean ± SD)	0.68 ± 0.19	0.58 ± 0.14	0.062

## 4 Discussion

### 4.1 Lateral wall considerations in surgical management

With the introduction of the lateral wall concept, the proximal femur has been redefined as consisting of five distinct components: (1) the femoral head–neck, (2) the femoral shaft, (3) the greater trochanter, (4) the lesser trochanter, and (5) the lateral wall (10). Anatomically, the lateral wall corresponds to the lateral femoral cortex distal to the vastus lateralis ridge, serving as a critical lateral buttress (11).

In contemporary fixation strategies for intertrochanteric fractures, whether intramedullary or extramedullary, screws traverse the lateral wall en route to the femoral head–neck. Thus, the integrity of the lateral wall is pivotal for construct stability. With proximal femoral nail antirotation (PFNA) devices, even in cases of intraoperative lateral wall fracture, the main intramedullary nail provides inherent support to the head–neck fragment and resists femoral shaft medialization (12), thereby partially compensating for loss of lateral wall integrity. By contrast, an intact lateral wall provides an essential lateral buttress for the cephalomedullary screw, enabling a three-point fixation construct that diminishes lever arm stress at the femoral head–nail interface, thereby reducing the risk of screw cut-out or nail fatigue failure.

From a biomechanical perspective, disruption of the lateral femoral wall eliminates a critical buttress against lateral migration of the proximal fragment and substantially increases varus collapse forces across the fracture site. Loss of this cortical support converts the construct from a stable three-point fixation model into a cantilever system, amplifying bending stresses on the cephalomedullary screw and the intramedullary nail. As a result, the load-sharing ability of the implant is markedly reduced, leading to excessive dynamic sliding, progressive medialization of the femoral shaft, and an increased risk of cut-out or implant fatigue failure. These mechanical consequences explain why lateral wall compromise is consistently associated with higher rates of fixation failure and reoperation in intertrochanteric fractures.

Multivariate regression analyses have demonstrated that lateral wall disruption is the strongest independent predictor of reoperation in intertrochanteric fracture fixation, exceeding even tip–apex distance in prognostic value (5). Therefore, in patients with intertrochanteric fractures and concomitant lateral wall involvement, intraoperative recognition of lateral wall instability warrants consideration of a long PFN supplemented with a lateral plate to restore buttress support and minimize the risk of implant migration.

It should be noted that the present study focused on preoperative fracture classification to analyze risk factors for lateral wall involvement; surgical techniques were not directly associated with the study outcomes. The most important clinical implication is meticulous intraoperative technique, particularly in patients with fracture lines less than 2 cm from the lateral wall margin, where inadvertent iatrogenic injury may convert an isolated intertrochanteric fracture into one complicated by lateral wall involvement.

The present findings extend current understanding of lateral wall involvement beyond the general concept of systemic osteoporosis. Although low BMD is a well-established risk factor for hip fractures, our results demonstrate that patients with concomitant lateral wall disruption exhibit a distinct pattern of skeletal fragility characterized by significantly reduced lumbar and overall hip BMD rather than localized deficits at the femoral neck or trochanteric region. This suggests that lateral wall compromise is not merely a by-product of osteoporotic bone but reflects a more advanced or diffuse cortical insufficiency that predisposes the lateral cortex to collapse under low-energy trauma. The observation that lower BMI independently predicted lateral wall involvement further supports the idea that reduced mechanical loading and diminished cortical robustness may specifically weaken the lateral column, making it more vulnerable than other regions of the proximal femur. Thus, our findings identify a unique clinical phenotype of intertrochanteric fractures in which systemic bone loss and reduced structural loading converge to directly influence the integrity of the lateral femoral wall.

### 4.2 Clinical significance of relevant indicators and analysis of risk factors for intertrochanteric + lateral wall femoral fractures

Bone mineral density (BMD): Thinning of the femoral lateral cortex and osteoporosis are recognized risk factors for lateral wall fractures in elderly patients (13). In a biomechanical study, Long et al. compared the use of the bridge combined fixation system (OBS) plus proximal femoral nail antirotation (PFNA) with PFNA alone for intertrochanteric fractures with lateral wall involvement, and reported that OBS reduced lateral wall stress by 46.9%, promoted safe bone healing, and obviated the need for stress shielding (14). Consistent with these findings, our study demonstrated that reduced lumbar spine and hip BMD were independent risk factors for lateral wall involvement. Prior investigations have further suggested that the occurrence and severity of osteoporotic intertrochanteric fractures are determined by systemic BMD decline rather than regional BMD at the hip, and that localized reductions do not directly correspond to site-specific fractures (15, 16). In our cohort, no significant between-group differences were observed in BMD at the femoral neck, Ward's triangle, or greater trochanter; however, overall hip BMD was significantly lower in patients with lateral wall involvement. Collectively, these results underscore that generalized BMD loss predisposes to lateral wall fractures, whereas localized BMD changes are not necessarily predictive. An additional consideration relates to the timing of BMD assessment. Because pre-injury or preoperative BMD measurements were not available, postoperative DXA values were used as a surrogate for baseline bone quality. Although BMD is unlikely to undergo meaningful change during the short interval between fracture, surgical fixation, and subsequent evaluation, postoperative measurement may not perfectly reflect true pre-injury skeletal status. Factors such as perioperative immobilization, acute inflammatory responses, or early postoperative metabolic changes could introduce minor deviations in measured bone density. Therefore, while postoperative DXA remains a widely accepted and practical method in hip-fracture research, the possibility of slight misestimation of baseline BMD should be acknowledged when interpreting its association with lateral wall involvement.

Age: Advancing age is associated with skeletal degeneration and senile osteoporosis. The Osteoporosis Self-Assessment Tool for Asians (OSTA) is a screening index inversely correlated with age, calculated as (weight−age) × 0.2; lower scores indicate higher risk of osteoporosis. Although patients with lateral wall involvement were significantly older than those with isolated intertrochanteric fractures, logistic regression revealed that age itself was not an independent predictor of lateral wall fractures.

Body mass index (BMI): BMI, calculated as weight in kilograms divided by height in meters squared, is the most widely used index of weight-for-height proportion. Low BMI has long been recognized as an indicator of reduced bone mass and impaired skeletal strength. Large-scale epidemiologic studies have demonstrated that lower BMI is strongly associated with decreased systemic BMD, even after adjusting for age and other covariates (17). This reduction in bone mass contributes to increased skeletal fragility, and multiple meta-analyses have confirmed that individuals with low BMI have a significantly higher risk of osteoporotic fractures (18). These findings align with prior orthopedic literature showing that patients with lower BMI often exhibit poorer bone quality, which may predispose them to structurally vulnerable fracture patterns, including those involving disruption of the lateral femoral wall (13). Low BMI is associated with osteoporosis and increased fracture risk. In our study, lower BMI was identified as an independent risk factor for lateral wall involvement. Patients with lower BMI were more likely to sustain lateral wall fractures in the setting of intertrochanteric injury, likely reflecting their reduced systemic BMD and greater susceptibility to osteoporosis.

Bone turnover markers: P1NP, a precursor of type I collagen synthesized by osteoblasts, reflects bone formation activity, whereas β-CTX, a degradation fragment of type I collagen, reflects osteoclast activity and bone resorption. These serum markers are widely regarded as reliable indicators of bone metabolism (19). Osteoporotic patients typically demonstrate reduced bone formation and/or enhanced bone resorption, reflected by decreased P1NP or elevated β-CTX. In our study, however, no significant intergroup differences were observed in either marker, indicating that bone turnover indices were not predictive of lateral wall fractures. The lack of significance may be explained by interindividual variability, non-normal data distribution, and the limited sample size.

## 5 Limitations

This study has several limitations. First, as a retrospective analysis, all enrolled patients had been admitted for surgical treatment and were therefore in relatively favorable health status, which may have introduced selection bias. Second, the stringent inclusion and exclusion criteria resulted in a relatively small sample size, which may have contributed to the non-normal distribution of bone turnover marker data. Future prospective, large-scale studies are warranted to validate and expand upon our findings. Additionally, because height and weight at the time of injury could not be directly measured, BMI values were obtained from prior medical records and verified through patient or family recall. This approach may introduce recall bias and reduce the accuracy of BMI assessment. Furthermore, this study was conducted at a single tertiary medical center, which may limit the generalizability of the findings to broader populations or different clinical settings. In addition, the relatively small sample size limits the statistical power of the regression analyses. Although the number of events per variable met basic methodological requirements for exploratory modeling, the study may still be underpowered to detect more subtle associations. Therefore, the findings—particularly regarding nonsignificant predictors—should be interpreted with caution and validated in larger prospective cohorts.

## 6 Conclusion

Increasing clinical evidence highlights the critical role of the lateral wall in maintaining stability following intertrochanteric fracture fixation. Intertrochanteric fractures with concomitant lateral wall involvement represent a distinct subtype in which inadequate management can adversely affect surgical outcomes and long-term prognosis. Identification of risk factors for lateral wall disruption is therefore essential to guide preventive strategies, including fall prevention and optimization of modifiable risk factors. In clinical practice, elderly patients of advanced age, with lower body mass index and reduced bone mineral density, should undergo meticulous preoperative evaluation of lateral wall integrity, with CT recommended when necessary to avoid missed diagnoses. Furthermore, patients with osteoporosis should receive timely anti-osteoporosis therapy to enhance bone quality and improve surgical outcomes.

## Data Availability

The raw data supporting the conclusions of this article will be made available by the authors, without undue reservation.
